# Editorial: Emergence and re-emergence of plant diseases caused by *Xanthomonas* species

**DOI:** 10.3389/fmicb.2022.1081601

**Published:** 2022-12-12

**Authors:** Teresa A. Coutinho, Marie-Agnes Jacques, Jeffrey Jones

**Affiliations:** ^1^Department of Biochemistry, Genetics and Microbiology, Centre for Microbial Ecology and Genomics/Forestry and Agricultural Biotechnology Institute, University of Pretoria, Pretoria, South Africa; ^2^Institut Agro, INRAE, IRHS, SFR QUASAV, University of Angers, Angers, France; ^3^Department of Plant Pathology, University of Florida, Gainesville, FL, United States

**Keywords:** geographic range, host range, virulence, aggressiveness, resistance

The emergence and re-emergence of plant diseases has increased noticeably in recent years. This has been attributed to climate change, monoculture and trade (Willocquet et al., 2020). An example is the occurrence of *Xylella fastidiosa* in different regions of the world (Almeida and Nunney, 2015). It was described as a “relatively obscure pathogen” in 2002 (Hopkins and Purcell, 2002), but 10 years later it is now considered a major threat to agriculture worldwide.

*Xanthomonas* species are members of the Lysobacteriaceae (previously known as Xanthomonaceae), a family within the class Gammaproteobacteria. They have an extremely wide host range, infecting both monocotyledonous and dicotyledonous plants. The number of first reports of novel *Xanthomonas* species or pathovars has increased in recent years (Bansai et al., 2022; Dia et al., 2022) as has the appearance of more aggressive strains of known species (Chen et al.). The same host has also been shown to harbor both pathogenic and non-pathogenic strains (Cesbron et al., 2015; Fernandes et al., 2021; Ramnarine et al., 2022). Furthermore, *Xanthomonas* species have been shown to have increased their host and geographic range (Curland et al., 2020; Sigillo et al., 2021). Horizontal gene transfer has been identified as one potential mechanism favouring host-range evolution (Chen et al., 2018).

*Xanthomonas* species can colonize novel hosts and inhabit unique ecological niches due to their extensive genomic diversity. This rich diversity has been shown to possibly be linked to different production systems (Abrahamian et al., 2019; Klein-Gordon et al., 2021) and cultivated regions (Chen et al., 2021). *Xanthomonas* species are also an important component of the plant, rhizosphere and soil microbiomes (da Silva et al., 2021). The ability of this species to infect novel hosts and occur in different regions of the world has led to an increased research on such topics as host-pathogen interactions, virulence and pathoadaption. There has also been an increased understanding of the ecology and epidemiology of these pathogens.

Differences in aggressiveness between strains of *X. campestris* pv. *campestris* to cabbage was recorded in China (Chen et al.). The genomes of aggressive and less aggressive strains were sequenced. Differences were noted in Type III secreted effector repertoires, virulence factors and secreted proteins. The authors thus found a direct correlation between genomic variability and virulence of *X. campestris* pv. *campestris*.

In China, two strains of *X. oryzae* pv. *oryzae* from two locations, low land and high mountain paddies, were shown to differ significantly in virulence (Li et al.). In a comparative genomic analysis, the strains differed in <10 genes, including a gene coding for the transcription activator-like effector (TALE). The *tal* genes were variable in both their number and sequence. This study thus highlighted the variability of TALEs genes in strains of *X. oryzae* pv. *oryzae*.

Bacterial spot, caused by four *Xanthomonas* species, is an important disease of tomatoes and peppers. Jibrin et al. identified a novel race (T5) in Nigeria which was shown to be the product of recombination between two of the four *Xanthomonas* species. Genomes of these species have only been sequenced from five African countries. The authors thus note that there is thus a lack of information on the evolution and biology of these pathogens on this continent which could provide a foundation for genome-informed host–pathogen and ecological interaction investigations that are novel to production systems in Africa.

## Author contributions

All authors listed have made a substantial, direct, and intellectual contribution to the work and approved it for publication.
